# Circulating Galectin-3 levels and Diabetic Nephropathy: a systematic review and meta-analysis

**DOI:** 10.1186/s12882-023-03226-x

**Published:** 2023-06-08

**Authors:** Yong Guo, Ling Li, Shanbiao Hu

**Affiliations:** 1 Clinical Research Center for Organ Transplantation in Hunan Province, Changsha, China; 2 Department of Organ Procurement Organization, The Second Xiangya Hospital of Central South University, Changsha, China; 3 Department of Urology, The Second Xiangya Hospital of Central South University, Changsha, China; 4 Department of Kidney Transplantation, The Second Xiangya Hospital of Central South University, Changsha, China

**Keywords:** Galectin-3, Diabetic nephropathy, Meta-analysis

## Abstract

**Aims:**

Changes of serum galectin-3 (Gal-3) is associated with the pathogenesis of diabetic nephropathy (DN). However, current literature indicates that the given results remain debatable and inconsistent. Hence, the aim of this present meta-analysis was to focus on the predictive role of serum Gal-3 in patients with DN.

**Methods:**

The PubMed, Embase, Cochrane Library and Web of Science databases were systematically searched for studies that reported the relationship between Gal-3 levels and DN risk, from the inception of each database to March, 2023. The literature we selected for inclusion based on inclusion and exclusion criteria. The standard mean difference (SMD) with corresponding 95% confidence intervals (95% CI) were used to investigate the association. When *I*^*2*^ value exceeding 50%, we will consider it has the presence of a higher level of heterogeneity. A sensitivity analysis and subgroup analysis were performed to seek the potential sources of heterogeneity. The quality assessment was performed using according to the Newcastle–Ottawa Quality Assessment Scale (NOS). The data analysis was conducted using STATA version 13.0 software.

**Results:**

We ultimately enrolled 9 studies enrolling a total of 3137 patients in the final analysis. The SMD of serum Gal-3 was higher in patients with DN group (SMD 1.10 ng/mL [0.63, 1.57]; *I*^*2*^: 96.1%). Upon removal of a study in sensitivity analysis, patients with DN had higher serum Gal-3 levels compared to control patients (SMD 1.03 ng/mL [0.52, 1.54], *I*^*2*^: 94.4%). Further subgroup analysis was performed based on the region. No matter in Asia, Europe or Africa, the serum Gal-3 level of DN patients is significantly higher than that of the control population (SMD: 0.73; 95% CI: 0.58 to 0.87 for Asian; SMD: 0.79; 95% CI: 0.48 to 1.10 for Europe; SMD: 3.15; 95% CI: 2.73 to 3.56 for Africa).

**Conclusion:**

In conclusion, these results suggested that higher serum Gal-3 may increase the risk of DN. More fundamental studies are necessary to clarify the exact physiopathological basis mechanisms of Gal-3 effects. In addition, further research, especially emphasis on the cut-off value should be given, and is best to predict their actual importance as well as the diagnostic accuracy.

**Supplementary Information:**

The online version contains supplementary material available at 10.1186/s12882-023-03226-x.

## Strengths and limitations of this study

1. To our knowledge, this is the first article to systematically evaluate meta-analysis for investigate the relationship between Galectin-3 and diabetic nephropathy.
2. Heterogeneity, subgroup analysis, sensitivity analysis, and publication bias were explored.
3. Studies included in this meta-analysis were conducted at various geographic locations, thus the overall effect may be generalizable.

## Introduction

Diabetic nephropathy (DN) is one of the most typical feature in diabetes. The clinical manifestations are persistent microalbuminuria, a progressive decline in renal function, and renal interstitial fibrosis [1]. The incidence of DN is around 25%-40% type 1 diabetes patients, and 5%-40% in those with type 2 diabetes [2]. Epidemiological data show that about 50% of DN patients will eventually develop into end-stage renal disease (ESRD) without effective therapy [3]. In addition, patients with DN accelerates the progression of coronary heart disease, stroke, and peripheral artery disease [4]. At present, the pathogenesis of DN has not been clearly clarified, and hyperglycemia may be one of the important factors for the occurrence of DN, causing kidney progressive damage either directly or through hemodynamic changes [5]. Relationships between DN and additional risk factors were examined, including hypertension, dyslipidemia, obesity, and smoking [6]. Renal hyperfiltration, renal injury, and glucose metabolic disorder has been involved in the pathogenesis of DN [7]. Chronic inflammatory response and oxidative stress play a major role in DN development and could be a useful means to avoid DN risks [8]. According to Mogensen Stage, DN progress can be further divided into five stages. Once developed into stage V, the disease would be difficult to deal with to treat for the complex metabolic disorders [9]. Currently, intensive glycemic control is the most useful treatment to treat DN, and thus the use of new antidiabetic drugs with renal protective effects are recommended for clinical use [10]. Several previous studies have shown that treatment with angiotensin converting enzyme inhibitors (ACEIs) or angiotensin receptor blockers (ARBs) slows kidney disease progression [11]. Despite the pharmaceutical drug industry progresses rapidly, the side effects of antidiabetic drugs have limited widespread clinical use of drug. Glomerular filtration rate (GFR) and microalbuminuria analyses have been proposed as predictors of long-term renal function, but they are often displayed both low specificity and sensitivity, and often not confirmed when more than 50% reduction in kidney function has occurred [12]. Moreover, despite an increasing number of molecule markers recognized as the degree of histopathologic changes in DN patients [13], their sensitivity and specificity are unsatisfactory. Therefore, identification of underlying novel molecular markers for early prediction of DN is vitally needed. In addition, still there is lacking efficient approaches for DN [14], and it seems that discovering the mechanism underlying disease could be a greatly beneficial for discovering novel treatment options. Thus, more useful biologic markers and exploring the disease’s underlying mechanisms should be seek for patients affected with DN.

Mammalian galactosides are a family of water-soluble sugar-binding proteins characterized by a carbohydrate-recognition domain with an affinity for β-galactosides [15]. Among the galectin family, Galectin-3 (Gal-3) is the most studied member of the galectin family. Gal-3 are a family of proteins rich in proline- and glycine. This domain is responsible for binding to β-galactosides on cellular. Gal-3 is located in the cytoplasm and the nucleus, which is produced by a variety of cells, including macrophages, vascular smooth muscle cells (VSMCs), and endothelium [16]. Gal-3 can be transported to the cell surface, extracellular space, and the circulation [17]. This protein is involved in diverse physiological and pathophysiological processes such as cell proliferation, apoptosis, differentiation, and tumor progression [18]. Gal-3 has been shown to activate the NF-κB signaling pathway, which is involved in the regulation of inflammation, and promote the production of pro-inflammatory cytokines [19]. The increased inflammatory response in the kidney may lead to the development of renal injury and proteinuria in diabetes. In addition, Gal-3 has been shown to be involved in the regulation of extracellular matrix production and deposition, which are key processes in the development of renal fibrosis [20]. The increased deposition of extracellular matrix components, such as collagen, may lead to glomerular basement membrane thickening and interstitial fibrosis, resulting in loss of renal function.

There is a growing interest in investigating the potential role of Gal-3 in the development and progression of DN, a common complication of diabetes. Several studies have reported elevated levels of galectin-3 in patients with DN, suggesting its potential as a biomarker for disease diagnosis and prognosis. However, the results of individual studies have been inconsistent, and a meta-analysis would be necessary to provide a more comprehensive understanding of the relationship between Gal-3 levels and DN. By pooling data from multiple studies, a meta-analysis would enable the identification of any patterns or trends that may not be apparent from individual studies alone. Furthermore, a meta-analysis could help to address some of the limitations of individual studies, such as small sample sizes and variations in study design and methodology. Ultimately, a meta-analysis of Gal-3 levels and DN would provide valuable insights into the potential use of Gal-3 as a biomarker for this common complication of diabetes.

## Methods

### Search strategy

This meta-analysis was conducted according to the Preferred Reporting Items for Systematic Reviews and Meta-analysis (PRISMA) statement [21]. In order to find relevant literature reported association between serum concentration of Gal-3 and DN, we systematically searched the four component databases: PubMed, Embase, Cochrane Library and Web of Science databases. English articles with no date restriction were searched. The search terms used were listed as follow: “Diabetic nephropathy”, “Galectin-3”. The search time ended in March 2023. Further, references list of included studies were also manually checked for relevant articles.

### Inclusion and exclusion criteria

The inclusion criteria in our meta-analysis were based on the following aspects: (1) serum concentration of Gal-3 was examined; (2) mean ± standard error of the mean (SEM) was reported for Gal-3 between patients with or without DN. Studies were excluded according to the following criteria: (1) duplicate studies; (2) nonoriginal research articles; (3) the research without required data; (4) involved nonhuman studies; (5) Excluding cardiovascular disease, inflammation, tumors such as breast cancer and colon cancer, obesity, aging, exposure to environmental toxins such as heavy metals and pesticides as well as dietary factors such as low levels of vitamin D, magnesium, and calcium, may affect the levels of Galectin-3. Studies were selected by two reviewers (YG and GL) for inclusion in our analysis using the aforementioned criteria, and disagreements were resolved by consensus or with the help of a third reviewer (SH).

### Data extraction

Data extraction of included studies were extracted independently by two persons. The following details were extracted: first author, year of publication, country of study population, age of patients, number of patients included in the study, detection method for serum Gal-3, expression of Gal-3 in serum with or without DN, and NOS scores.

### Quality assessment

We assessed the methodological quality of included study using the Newcastle–Ottawa Quality Assessment Scale (NOS) for non-randomized studies [22]. A study with NOS above 5 was categorized as a high-quality of research [23]. NOS, which ranges from 1 to 9 stars and judges each study regarding the following three aspects: the selection of the study groups, the comparability of the groups, and the ascertainment of the outcome of interest.

### Statistical analysis

All statistical analyses were performed in the meta-analysis using STATA version 13.0 (Stata Corp LP, College Station, TX) software. The results for the association between Gal-3 and the DN was evaluated using standard mean difference (SMD) and 95% confidence interval (CI). *I*^*2*^ was used to evaluate statistical heterogeneity among the studies. The fixed-effects or random-effects models were used according to the *I*^*2*^ value (*I*^*2*^ < 50%, fixed-effects models; *I*^*2*^ > 50%, random-effects models). Meta-analysis results will be graphically presented on a forest plot. Subgroup analysis was carried out according to different region (Africa vs Europe vs Asia). Statistical significance was considered when the bidirectional *p* value less than 0.05. We performed sensitivity analysis to evaluate whether the combined results were stable and reliable, and used the Egger's and Begg's test to evaluate the potential publication bias.

## Results

### Identification of studies

**Fig. 1:**
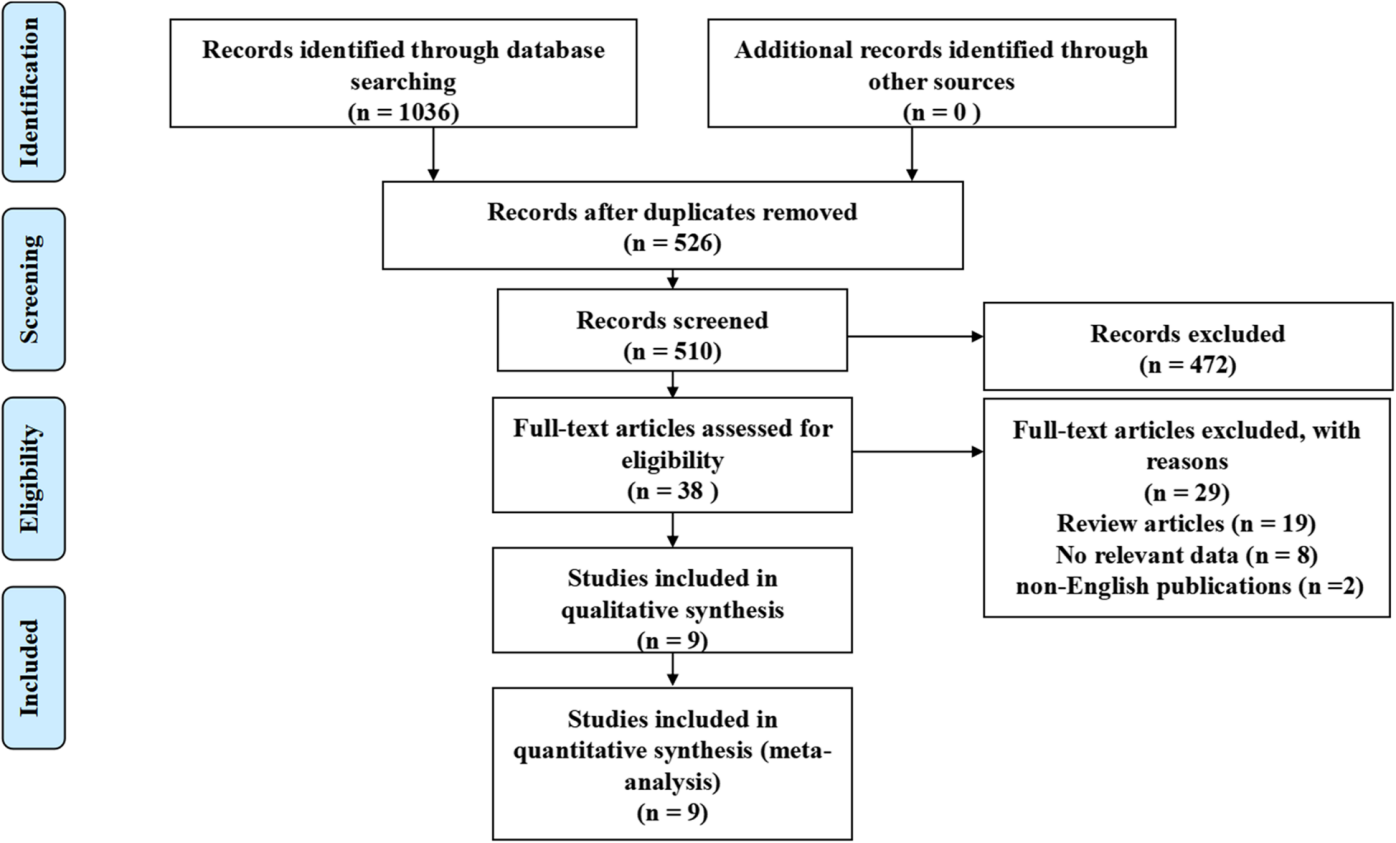
Flow of study selection

Following the systematic search strategy, 1036 papers were retrieved via the 4 databases. 526 studies remained after removing duplicates. 472 studies were excluded by comprehensively screening the titles, abstracts. The full texts of 38 remaining studies were reviewed. Of them, 19 were further excluded because of review articles, 8 did not show relevant data, and the other two were non‐English publications. Eventually, 9 studies [24–30] consisting of 3137 patients that were published between 2013 and 2021 were included in our meta-analysis. The details of flow diagram of literatures screening were presented in Fig. 1.

### Study characteristics

The quality assessments of each included article were presented in Table 1 and the main detailed characteristics of included studies were summarized in Table 1. The year of publication period ranged from 2013 to 2021. The mean ages of the patients in all included studies varied between 46.7 and 68 years. The countries in which these studies occurred included the China (*n* = 3), Iraqi (*n* = 1), Egypt (*n* = 1), Germany (*n* = 1), India (*n* = 1), Italy (*n* = 1), and Turkey (*n* = 1). The levels of Gal-3 levels were measured enzyme linked immunosorbent assay (ELISA) in most of the included studies (8/9). Overall, the included studies were generally of good quality, with the NOS scores counted from 6 to 9.

**Table 1:** Characteristics of available studies relating Gal-3 levels to DN risk

**Number**	**Author**	**Year**	**Age**	**Country**	**Assay**	**Diagnostic criteria for DN**	**Disease condition**	**DN**	**Control**	**NOS**
1	Kathryn C. B. Tan	2018	52.4±5.2/52.0±3.2	China	ELISA	eGFR, 60 ml/min per 1.73 m2	eGFR< 60 ml/min per 1.73 m2	7.58±2.29	6.10±1.91	8
2	Nihal Yücel	2016	58.71±9.97/63.26±10.75	Turkey	ELISA	NA	Microalbumin / Creatinine (mg/gr) > 300	8.68±2.41	7.78±2.05	7
3	Christiane Drechsler	2015	62.8±10.5	Germany	ELISA	eGFR, 60 ml/min per 1.73 m2	eGFR< 60 ml/min per 1.73 m2	23.16±9.9	12.86±4.0	8
4	Salman Hussain	2020	56.2±11.34/56.2±10.39	India	ELISA	eGFR, 90 ml/min per 1.73 m2; macroalbuminuria (UACR > 30mg/day).	UACR > 300mg/day	15.1±12.19	8.3±7.15	8
5	Massimo Iacoviello	2016	68±12/ 62±13	Italy	NA	urinary albumin/creatinine ratio (UACR) > 30 mg/g	UACR > 30mg/day	19.9±8.8	14.6±5.5	6
6	JIN Qi-hui	2013	46.7±13.4/48.7±15.0	China	ELISA	albumin excretion of ≥ 30 mg/d	albumin excretion of ≥ 30 mg/d	27.4 (10.9,72.7)	17.6 (5.1,39.4)	7
7	hossam hodeib	2019	54.93±7.13/55.57±4.36	Egypt	ELISA	ACR > 30 mg/g	ACR >300 mg/g	19.15±2.85	9.02±3.55	7
8	Gaofeng Song	2018	53.2±11.4/63.7±9.6	China	ELISA	Mogensen DN diagnostic criteria	eGFR< 15 ml/min per 1.73 m2	5.92 (4.66-7.35)	2.40 (1.69-3.39)	9
9	Najlaa abed jassim	2021	53±6.06/55.03±4.9	Iraqi	ELISA	ACR >30 mg/g	ACR >300 mg/g	3.002±1.74	1.60±0.62	8

*ELISA* Enzyme-Linked Immunosorbent Assay, *DN* Diabetic nephropathy, *UACR* Urinary albumin/creatinine ratio, *eGFR* estimated glomerular filtration rate, *ACR* Albumin-to-Creatinine Ratio

### Meta-analysis

**Fig. 2:**
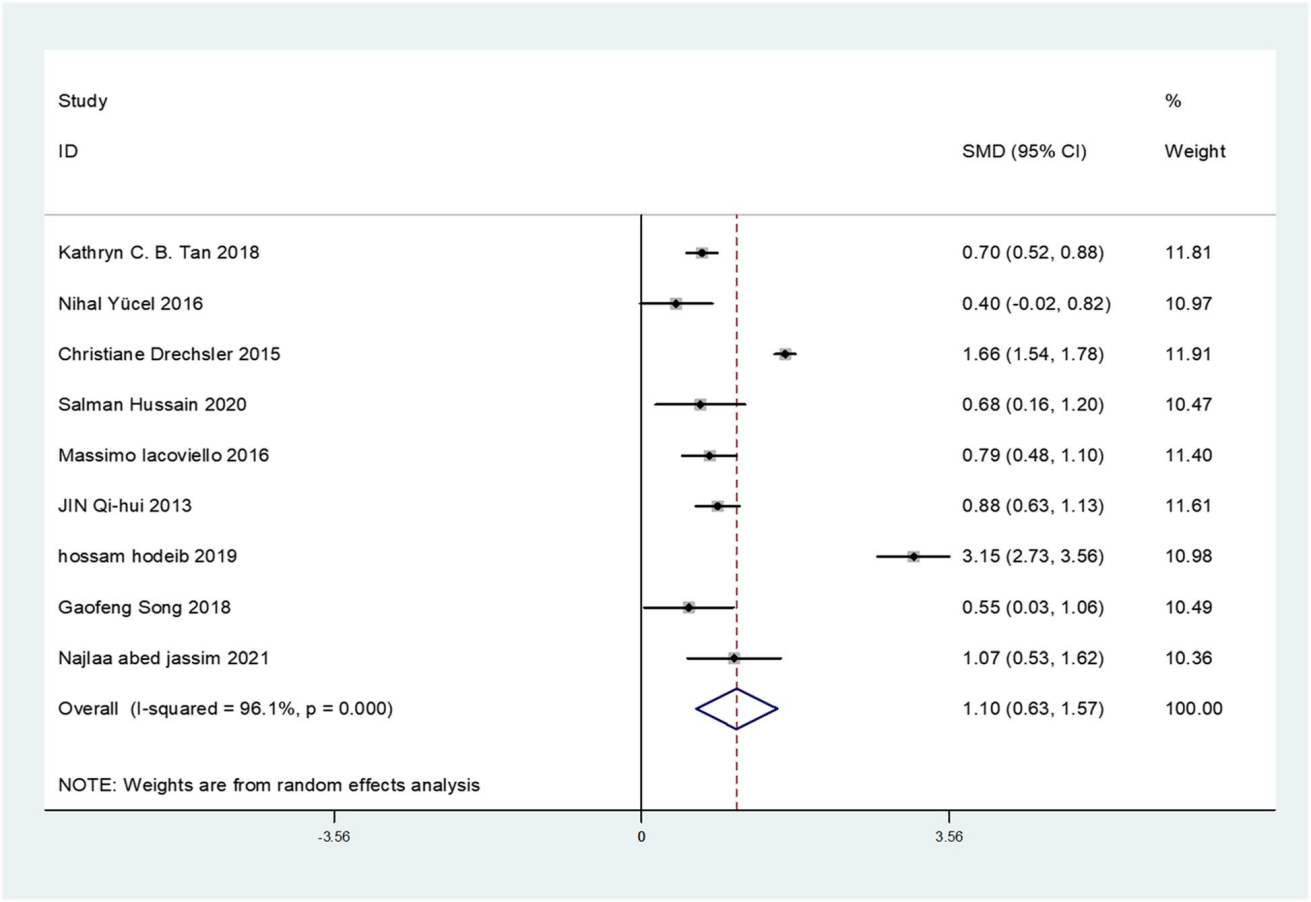
Forest plot evaluating Gal-3 levels in people with DN versus those without DN. The pooled results are expressed as SMD with their 95% CI

**Fig. 3:**
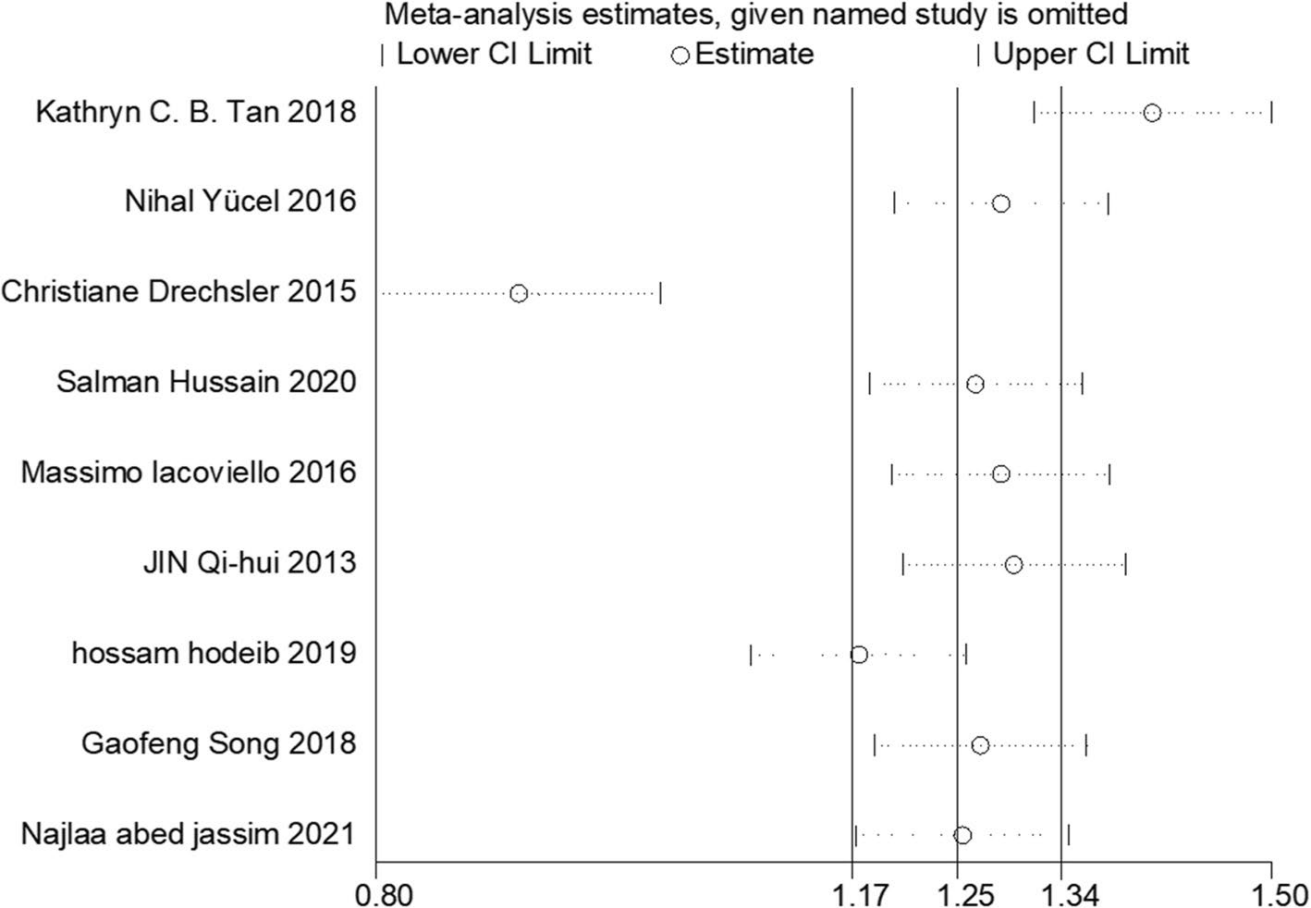
Sensitivity analysis for publication bias. Sensitivity analysis for the effects of Gal-3 levels on DN

**Fig. 4:**
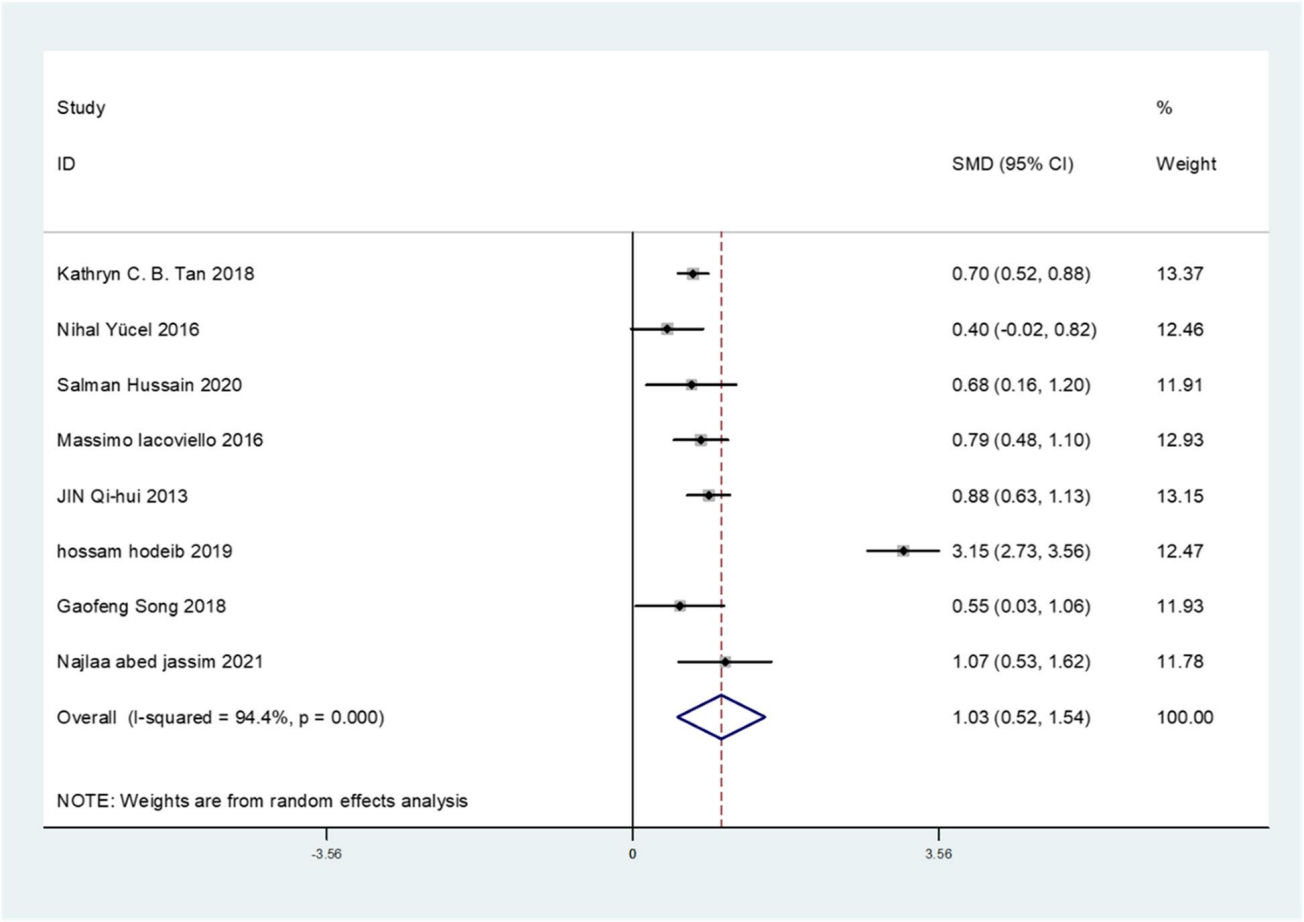
Forest plot evaluating Gal-3 levels in people with DN versus those without DN after removing one study. The pooled results are expressed as SMD with their 95% CI

**Fig. 5:**
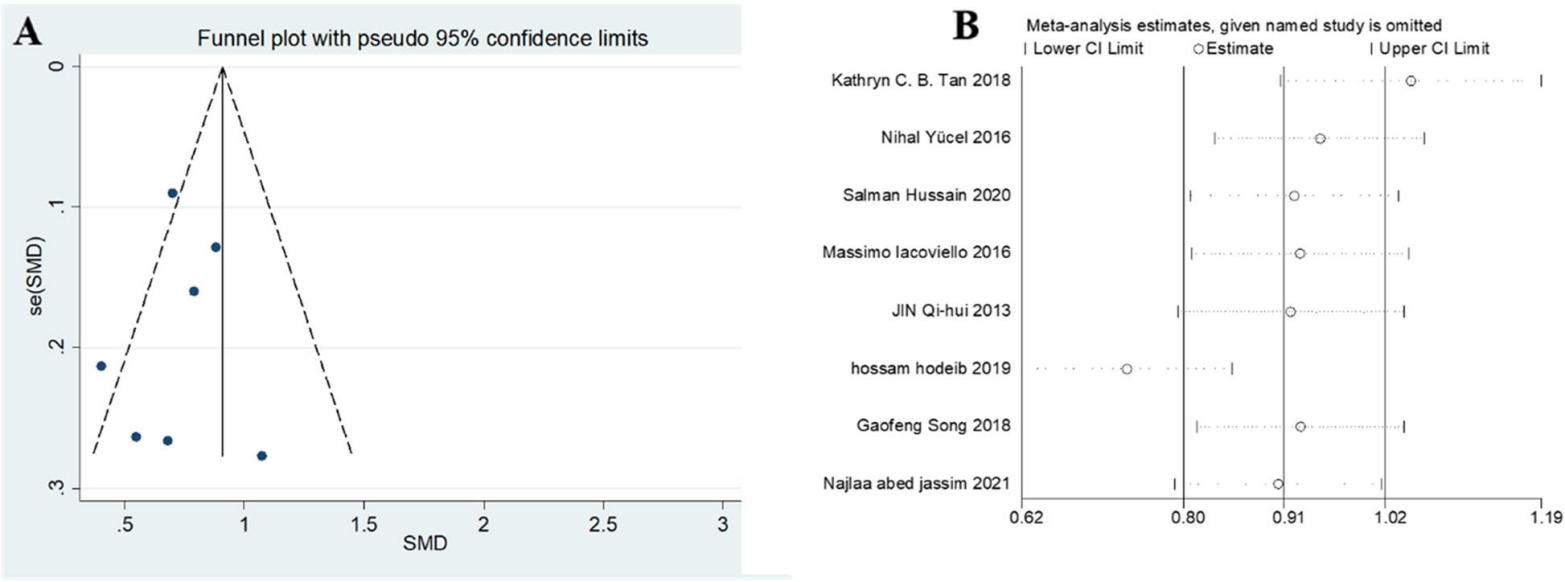
Funnel plots (**A**) and Sensitivity analysis (**B**) and for publication bias after removal of the one study

All of the included 9 studies reported circulating Gal-3 levels in patients with or without DN. Due to the statistical significance of heterogeneity among these studies (*I*^*2*^ = 96.1%; *p* < 0.001), we used a random-effect model to calculate the pooled SMD and 95% CI. Pooled results showed that circulating Gal-3 levels were significantly higher in patients who with DN compared to patients who without DN (SMD: 1.10; 95% CI: 0.63 to 1.57, *I*^*2*^ = 96.1%) (Fig. 2). To evaluate the effect of individual study in DN risk, we used a sensitivity analysis by successively omitting each individual study from the pooled analysis (Fig. 3). We found that one article substantially influenced the results in this analysis. After removing studies according to sensitivity analysis, 8 studies remained to be meta-analyzed. These results suggest that patients who developed DN had higher circulating Gal-3 levels compared to those who did not develop DN (SMD: 1.03; 95% CI: 0.52 to 1.54, *I*^*2*^ = 94.4%) (Fig. 4). Due to the heterogeneity, a subgroup analysis was conducted to figure out the source of heterogeneity. In term of region, high expression of Gal-3 is significantly related to DN risk (SMD: 0.73; 95% CI: 0.58 to 0.87 for Asian; SMD: 0.79; 95% CI: 0.48 to 1.10 for Europe; SMD: 3.15; 95% CI: 2.73 to 3.56 for Africa) (Table 2). These results revealed that region was significant factors for the heterogeneity of the meta-analysis result. The funnel plot showed funnel asymmetry, indicating that a certain extent of publication bias possibly existed (Fig. 5). Begg’s test and Egger’s test were performed to verify whether there was publication bias in this meta-analysis. The *p* values were greater than 0.05 (Begg’s *p* = 0.536 and Egger’s *p* = 0.535), indicating that there was no significant publication bias in our meta-analysis.

**Table 2:** Subgroup analysis by region of measurement of Gal-3 levels in patients with DN and non-diabetic nephropathy (NDN)

Parameters	Subgroup	Study	SMD	95%CI	12
Galectin-3	Asian	6	0.73	(0.58, 0.87)	15.60%
	Europe	1	0.79	(0.48, 1.10)	/
	Africa	1	.015	(2.73, 3.56)	/

## Discussion

**Fig. 6:**
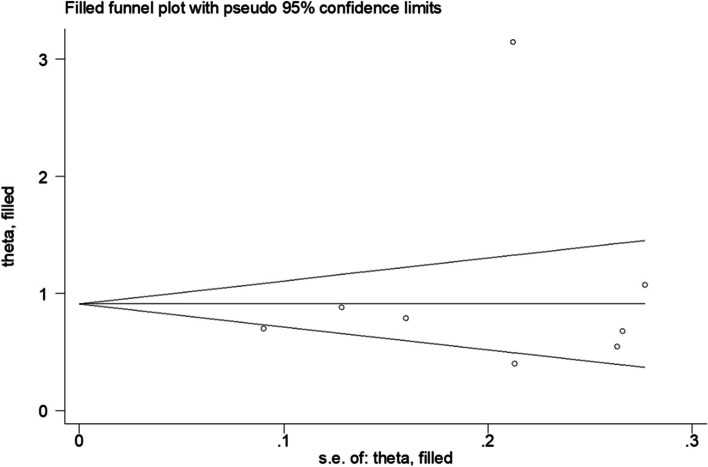
Trim-and-fill analysis of the studies enrolled

To our knowledge it is the first meta-analysis to report the results of circulation of Gal-3 and DN risk. Some studies have reported the relationship between Gal-3 expression and DN risk; however, the actual impact of Gal-3 expression on DN risk is still under discussion as the results of individual studies vary. In the present meta-analysis, we reviewed published studies, summarized existing evidence and performed a meta-analysis to generate a more accurate estimate of the predict value of Gal-3 for DN. Our meta-analysis combined the outcomes of 3137 patients from 9 studies, and indicated that the high level of Gal-3 shows a significant association for DN (SMD = 1.03, 95%CI = 0.52–1.54). Consequently, we operated subgroup analysis to analyze the source of heterogeneity. Subgroup analyses further confirmed that region significantly influence the results, suggesting different region may be the cause of heterogeneity. Although risks of publication biases were detected for DN risk, Beggs test, Eggers test and trim-and-fill analyses showed significant associations between higher serum Gal-3 on admission and higher risk of DN (Fig. 6). The stability of the pooled results was evaluated by sensitivity analysis (Fig. 5B), which indicated that the results were stable. In other words, higher level of Gal-3 may be associated with DN risk.

Our results have some significant clinical implications. Firstly, our findings supported use of serum Gal-3 level as a risk factor for DN. Secondly, this study adhered to PRISMA guidelines, which ensured methodological rigour and transparency. Thirdly, serum Gal-3 levels were quantitated via ELISA in 8 of the studies, and therefore, we consider that the values are comparable. The strength of this study was comprehensive search strategy with a thoroughly performed quality assessment. Moreover, this is the first meta-analysis to examine the role of Gal-3 for DN, which could provide some references for clinical doctors. Finally, the combined data of articles from different countries/regions were included, thus the overall effect may be quite general. However, it is important to note some limitations. Firstly, one of the primary limitations of meta-analyses on Gal-3 in DN is the need to determine a critical threshold for Gal-3 levels. However, different studies have used varying Gal-3 thresholds, making it difficult to establish a consensus on the predictive value of Gal-3 for DN development. Thus, additional clinical studies are required to validate these thresholds and to determine their clinical utility. Overall, further research is needed to fully understand the potential of Gal-3 as a biomarker for DN. Secondly, after conducting a subgroup analysis, meta-analyses may still show heterogeneity, which could be related to patient characteristics. Future studies could focus on recruiting more homogeneous patient populations to reduce the potential impact of patient characteristics on study outcomes. By addressing these issues, meta-analyses can improve the accuracy and reliability of their findings, leading to better clinical decision-making. Thirdly, one of the few papers discusses the concentration range in DN patients. However, the results were not appropriate from the retrieved study subjects due to the differential expression patterns across different geographical locations, which cannot hold the steady Gal-3 concentration. Finally, but no less important, it should be noted that its sample size is limited. Due to these limitations, the findings from this study should be interpreted with caution, and further research is needed to confirm the results of this meta-analysis. However, our study results provide the most robust evidence to date for the predictive role of Gal-3 in the development of DN. As such, this meta-analysis highlights the importance of continued research into the potential use of Gal-3 as a biomarker for DN, and underscores the need for more large-scale studies to validate these findings.

The potential mechanism of Gal-3 in DN requires further research. A number of possible reasons could account for this. First of all, Gal-3 expression is widespread throughout the body, including in the heart, brain, and blood vessels [31], and an important in inflammatory and proliferation [32]. Gal-3 promote inflammatory responses through activating macrophage, and predicts cardiovascular disease (CVD). As the main regulator of pro-inflammatory agent, Gal-3 was critical for neutrophil killing during endothelial-neutrophil interaction [33] and participate in wide variety of immune responses and activating numerous cell types [34] which ultimately leading to fibrosis. This is essential for the direct repair of injured tissues such as cardiac and renal. There is growing evidence that inflammation-induced vascular injury of diabetic patients could be the mechanism that contributing to increased susceptibility of vascular complications [35]. Gal-3 could be acting as an inflammatory factor in vascular disease, and thereby accelerating adverse ventricular remodelling [36]. Otherwise, it has also been hypothesized that Gal-3 may reduce oxidative stress indirectly by degrading advanced glycation end products (AGEs), leading to lower levels of HbA1c [37]. Gal-3 is a component of the AGE-receptor complex and is involved in the elimination of these pathogenic compounds [38]. AGEs have been shown to induce the expression of Gal-3 in cultured endothelial cells and within renal tissues in the diabetic milieu [39]. Gal-3 could bind to AGEs, stimulating their degradation [40], but the effect of Gal-3 in reducing HbA1c has not been previously reported. The exact mechanism is worth exploring in our further study.

## Conclusion

In conclusion, it was the first time for us to revealed the predict role of elevated Gal-3 expression in DN. Our meta‐analysis indicates that Gal-3 could be a biomarker for predicting the patients with DN. Further studies with larger sample size and well-design are needed to confirm the result. The clarification of the function of Gal-3 helps us to further understand the mechanisms of DN initiation and progression.

## Supplementary Information

**Additional file 1.**

## Data Availability

All data generated or analysed during the present study are included in this published article.
