# Correction to: Circulating galectin-3 levels and diabetic nephropathy: a systematic review and meta-analysis

**DOI:** 10.1186/s12882-023-03274-3

**Published:** 2023-07-26

**Authors:** Yong Guo, Ling Li, Shanbiao Hu

**Affiliations:** 1 Clinical Research Center for Organ Transplantation in Hunan Province, Changsha, China; 2 Department of Organ Procurement Organization, The Second Xiangya Hospital of Central South University, Changsha, China; 3 Department of Urology, The Second Xiangya Hospital of Central South University, Changsha, China; 4 Department of Kidney Transplantation, The Second Xiangya Hospital of Central South University, Changsha, China

**Correction to: BMC Nephrology (2023) 24:163**

10.1186/s12882-023-03226-x

Following publication of the original article [1], we have been informed that the first author has the first name and last name interchanged.

The author group has been updated above and the original article has been corrected.
